# A transfer learning approach based on gradient boosting machine for diagnosis of Alzheimer’s disease

**DOI:** 10.3389/fnagi.2022.966883

**Published:** 2022-10-05

**Authors:** Mehdi Shojaie, Mercedes Cabrerizo, Steven T. DeKosky, David E. Vaillancourt, David Loewenstein, Ranjan Duara, Malek Adjouadi

**Affiliations:** ^1^Department of Electrical and Computer Engineering, Center for Advanced Technology and Education, Florida International University, Miami, FL, United States; ^2^Fixel Institute for Neurological Disorders, University of Florida, Gainesville, FL, United States; ^3^1Florida ADRC (Alzheimer’s Disease Research Center), University of Florida, Gainesville, FL, United States; ^4^Department of Applied Physiology and Kinesiology, University of Florida, Gainesville, FL, United States; ^5^Center for Cognitive Neuroscience and Aging, Miller School of Medicine, University of Miami, Miami, FL, United States; ^6^Wien Center for Alzheimer’s Disease & Memory Disorders, Mount Sinai Medical Center, Miami, FL, United States

**Keywords:** Alzheimer’s disease, transfer learning, machine-learning, classification, gradient boosting machine, data distribution

## Abstract

Early detection of Alzheimer’s disease (AD) during the Mild Cognitive Impairment (MCI) stage could enable effective intervention to slow down disease progression. Computer-aided diagnosis of AD relies on a sufficient amount of biomarker data. When this requirement is not fulfilled, transfer learning can be used to transfer knowledge from a source domain with more amount of labeled data than available in the desired target domain. In this study, an instance-based transfer learning framework is presented based on the gradient boosting machine (GBM). In GBM, a sequence of base learners is built, and each learner focuses on the errors (residuals) of the previous learner. In our transfer learning version of GBM (TrGB), a weighting mechanism based on the residuals of the base learners is defined for the source instances. Consequently, instances with different distribution than the target data will have a lower impact on the target learner. The proposed weighting scheme aims to transfer as much information as possible from the source domain while avoiding negative transfer. The target data in this study was obtained from the Mount Sinai dataset which is collected and processed in a collaborative 5-year project at the Mount Sinai Medical Center. The Alzheimer’s Disease Neuroimaging Initiative (ADNI) dataset was used as the source domain. The experimental results showed that the proposed TrGB algorithm could improve the classification accuracy by 1.5 and 4.5% for CN vs. MCI and multiclass classification, respectively, as compared to the conventional methods. Also, using the TrGB model and transferred knowledge from the CN vs. AD classification of the source domain, the average score of early MCI vs. late MCI classification improved by 5%.

## Introduction

Alzheimer’s disease (AD) is a progressive cognitive disorder that impairs memory, thinking, and language. Due to the aging population, the number of individuals living with AD is steadily increasing, and AD has become one of the most prevalent neurodegenerative diseases (Barnes and Yaffe, 2011). Thus, it is essential to develop efficient, noninvasive, and scalable practices for its diagnosis. Many studies have focused on the early stage of AD, e.g., Mild Cognitive Impairment (MCI), to detect potential AD patients and enable early intervention and optimal treatment (Ye et al., 2012). Among MCI patients, two stages of the disease can be distinguished; early mild cognitive impairment (EMCI) and late mild cognitive impairment (LMCI), and the focus of studies is on detecting the EMCI stage. To measure the biomarkers of AD, there are several neuroimaging modalities, including magnetic resonance imaging (MRI), functional MRI (fMRI), computed tomography (CT), and positron emission tomography (PET) (Mehmood et al., 2021).

In various fields, such as computer vision, natural language processing, and speech recognition, machine learning has achieved promising advances. In the medical domain, machine learning is being employed to create a supplementary tool for disease diagnosis (Lee et al., 2018; Shojaie et al., 2021). However, collecting and labeling medical data is costly and labor-intensive. Thus, some medical datasets may not have a sufficient amount of data required for training a robust machine learning model. On the other hand, combining various datasets may introduce new challenges such as differences in the marginal and conditional distribution of the data due to disparity in imaging machines and tools, data collection policies, and characteristics of the participants. In machine learning, it is often assumed that the feature and label spaces and their distribution are the same for the training and testing datasets. If the data distribution is not the same, a trained learner on a source dataset may perform poorly on a target dataset. On the other hand, the labeled data of the target domain may not be enough to train a separate model. To handle this challenge, transfer learning is used to effectively transfer the information between domains considering data distribution discrepancy. This will make it possible to build predictive models in a target dataset with a limited amount of data (Zhuang et al., 2021). In general, transfer learning can be used in a scenario where there is a link between the two learning tasks. If the connection between the domains is misleading or misinterpreted by the model, negative transfer may occur where transferring information may have a negative impact on the target learner (Wang et al., 2019).

In terms of solution, transfer learning approaches can be classified into instance-based, feature-based, or model-based techniques. Instance-based approaches focus on transferring knowledge through the source domain instances while using a weighting scheme as a filtering criterion. In feature-based transfer learning, the source features are transformed, and a new feature space is built. The aim of the model-based transfer learning is to take a pre-trained model built on a primary dataset and fine-tune and use it for a smaller dataset (Pan and Yang, 2010). As an instance-based method, TrAdaBoost, which is an extension of the AdaBoost algorithm, has been presented in Dai et al. (2007). In this iterative approach, the training data of the source and target domains are combined, and a model is trained. In each iteration, if an instance from the target domain is misclassified, a higher weight is assigned to it, while the misclassified instances from the source domain will receive a lower weight for the next step. Thus, the different-distribution training instances from the source domain will have a lower impact on the final model. An extended version of TrAdaBoost for multi-source transfer learning scenarios has been developed in Yao and Doretto (2010) and is called MsTrAdaBoost. Another multi-source transfer learning (MSTL) approach has been proposed in Zhang et al. (2014) to facilitate the utilization of knowledge from multiple source domains. In Yang et al. (2020), a multi-source ensemble transfer learning (METL) approach is presented, which consists of a single-source tri-transfer learning and a multi-source ensemble learning.

Deep learning models such as convolutional neural networks (CNNs) have been extensively used for the automatic extraction of discriminative features and classification. Since deep learning requires a massive amount of data points, they are ideal candidates for transfer learning (Yang et al., 2020). For such situations, a part of the network will be transferred in a model-based manner. In a group of studies, the convolutional layers of feature extraction that are pre-trained on a general dataset are transferred for a specific task of disease diagnosis. In contrast, the classifier and fully connected layers are designed and trained for the desired classification task (Shin et al., 2016; Aderghal et al., 2018; Maqsood et al., 2019; Khan et al., 2019; Mehmood et al., 2021). Another approach to realize model-based transfer learning is to add a pre-trained model from a source domain to the objective function of a target learner and transfer the source knowledge during the target training procedure. A version of this approach has been proposed in a Domain Adaptation Machine (DAM) in Duan et al. (2012).

In this paper, a transfer learning framework is presented for building classification models for a target dataset with a limited amount of labeled data. For this purpose, a dataset with a sufficient amount of data is used as the source for transferring knowledge. The proposed approach uses the boosting mechanism to penalize source instances with different distributions than the target data. The framework is built on top of the GBM algorithm, and the residuals of the GBM base learners are used for defining weights for the source and target instances. A step-by-step description of the proposed algorithm is presented. To evaluate the effectiveness of the model, a set of experiments are conducted. The model performance is compared to two baseline scenarios where a model is trained solely based on either the source data or the target data. The results support the satisfactory performance of our boosting-based transfer learning model for multimodal multiclass classification in a source and target domain setup with different distribution of feature and label spaces.

## Data domains for transfer learning

In the transfer learning scenario, if the target domain does not include any label information, it is known as transductive learning, while if labeled data is available, the problem is referred to as inductive transfer learning (Pan and Yang, 2010). Transfer learning approaches can also be explained based on the degree of consistency between the target and source domains. Let XS and XT be the feature space of the source and target domains, and YS and YT be the label spaces, respectively. The subscripts S and T refer to the source and target domains. The data of the two domains may vary in different aspects. When the feature spaces or the label spaces are different (XS ≠ XT, YS ≠ YT), the problem is known as heterogeneous transfer learning. In contrast, homogeneous transfer learning is the case when the source and target domains have the same features and labels (Zhuang et al., 2021). For instance, in the AD classification, if different regional features or different modalities are used for the source and target domains, we are dealing with a heterogeneous transfer learning problem. Alternatively, the feature spaces could be the same, but their marginal distribution can be different (P(XS) ≠ P(XT)). Some studies only try to mitigate this discrepancy between the marginal distributions, which is known as sample selection bias (Huang et al., 2006; Sugiyama et al., 2008). As an example, a medical dataset may only include completely healthy subjects, while another dataset might have a minimum requirement of cognitive concern even for the cognitively normal group. Similarly, for the AD group, different datasets may differ in the severity of the biomarkers and the disease progression. These situations can lead to a shift in the distribution of the input data.

The other transfer learning scenario is when the source and target domains have different conditional probability distributions (P(YS| XS) ≠ P(YT| XT)). This condition is known as context feature bias. In this case, given identical input features, the target variable differs for the two domains. In the AD context, subjects with similar biomarkers may be mapped to different diagnosis groups due to various reasons. This can be partially due to the subjective procedure of data annotation and also heterogeneity of the disease, which can be explained by the AD subtypes and the risk and protective factors. For instance, in an AD group, younger subjects tend to have different levels and patterns of biomarkers than older ones, and if age is not considered, those subjects might be mapped to the wrong cognitive group.

Besides the marginal and conditional probability distributions of the input X, there can be a mismatch between the class space for the source and target domains (YS ≠ YT). The label space of the two domains may vary in terms of the number of classes and the class labels themselves. In our study, the source domain might be dealing with a binary classification between the CN and AD groups, while the target domain may focus on a multiclass classification or a binary classification with different labels such as early MCI and late MCI. The other scenario is when the data is unbalanced between the two sources resulting in a disparity between the probability distribution of the labels (P(Ys) ≠ P(YT)). In the ADNI dataset, there is a lower number of AD subjects compared to CN and MCI, while the Mount Sinai dataset has balanced AD and CN but more MCI subjects. In this study, the feature space of the two datasets is the same, while their marginal and conditional distributions can be different. As for the label space, there is a mismatch between the class labels and their distributions in some scenarios.

## Data and methods

### Data

For our analysis, the clinical data were obtained from two datasets. For the target domain, the participants are part of the 1Florida Alzheimer’s Disease Research Center (ADRC) in a 5-year study since 2015 at the Mount Sinai Medical Center. The number of subjects for each group of CN, MCI, and AD in this dataset is 53, 141, and 45, respectively. As part of the 1Florida ADRC baseline analysis, a wide range of neuropsychological and clinical tests were performed, as well as neuroimaging studies such as structural MRI and PET/CT scans to measure fibrillar amyloid plaques. Each participant underwent clinical assessments, including the Clinical Dementia Rating (CDR) and the Mini Mental State Examination (MMSE). The neuropsychological examination also incorporates the Hopkins Verbal Learning Test-Revised (HVLT-R). For each participant, structural MRI was acquired at Mount Sinai Medical Center using a Siemens Skyra 3T MRI scanner. The FreeSurfer software was used for brain segmentation using a 3D T1-weighted sequence (MPRAGE) with isotropic resolution of 1.0 mm. A 3D Hoffmann brain phantom was used for PET scan acquisition. PET tracer [18-F] florbetaben 300 MBQ was infused 70–90 min before scanning. Each subject was scanned on a Siemens Biograph 16 PET/CT scanner (55 slices/frame, 3 mm slice thickness, 128 × 128 matrix). The scans were then transformed to a 128 × 128 × 63 dimension with the size of 0.21 cm × 0.21 cm × 0.24 cm.

On the other hand, the Alzheimer’s Disease Neuroimaging Initiative (ADNI) database^1^ was used as the source domain. ADNI was launched in 2003 as a public-private partnership, directed by Principal Investigator Michael W. Weiner, MD. The primary objective of ADNI has been to test whether serial MRI, PET, other biological markers, and clinical and neuropsychological assessments can be combined to measure the progression of MCI and early AD. For up-to-date information, see http://www.adni-info.org. The participants’ demographics and Mini-Mental State Examination (MMSE) scores for ADNI 3 cohort used in this study are given in Table 1. The modalities MRI and amyloid PET (agent: ^18^F-AV45) for one subject visit were included in the analysis.

**TABLE 1:** Participant demographics and mini-mental state examination (MMSE) score for different diagnosis groups of the ADNI3 cohort.

Groups	Subject (f/m)	Age (year)	Education (year)	MMSE
CN	277 (153/124)	71.80 ± 5.70	16.67 ± 2.47	28.63 ± 2.12
MCI	378 (155/223)	71.26 ± 7.66	16.25 ± 2.61	26.87 ± 4.20
AD	67 (26/41)	73.41 ± 8.78	16.43 ± 2.35	22.37 ± 2.39

The T1 weighted MRI scans have gone through preprocessing, gradient wrapping, scaling, shading artifact, and inhomogeneity corrections. For skull stripping and cortical and subcortical segmentation of the T1 images, the FreeSurfer package was used. The segmented MRI scans were then co-registered with the florbetapir scans to measure the volume-weighted average of amyloid deposition in regions of interest and compute the standardized uptake value ratio (SUVR). Besides these neuroimaging biomarkers, AD risk and protective factors, including age, gender, APOE4, and education, are also used as independent variables for each subject. These variables, when combined with the biomarkers, can help with the disease heterogeneity challenge.

### The proposed transfer learning approach

**FIGURE 1:**
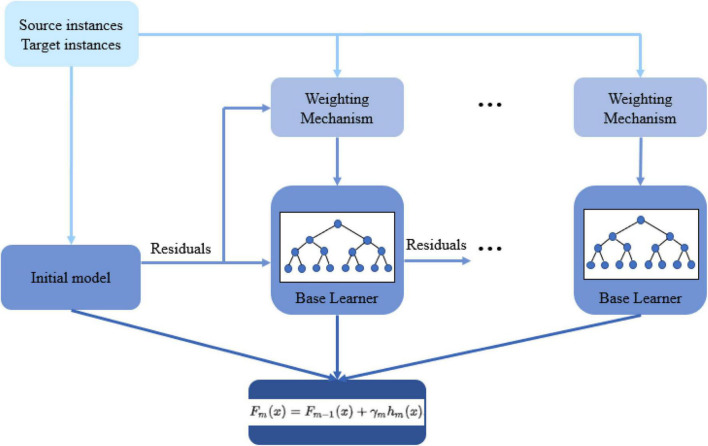
The structure of the proposed gradient boosting-based transfer learning approach (TrGB).

A source domain can contribute to a target domain learner directly through transferring the data. This category of transfer learning is known as instance-based transfer, which is the base of the approach of this study. Initially, the source data is added to the training set since the labeled target data is usually not enough to train an effective model. The main idea is to take advantage of the same-distribution portion of the source data and gradually fade the effect of the misleading part with a different distribution. To do so, we present an extended version of GBM for transfer learning (TrGB). GBM, which was first introduced in Friedman (2001), is a boosting-based learning algorithm that combines a sequence of base learners. Each base learner focuses on the error (residuals) of the previous learner, and this process is repeated until the error is less than a predefined threshold. A final prediction is made based on the combination of the response of all learners. The overall model variance is low because of using simple base learners, and a low bias is achieved through the boosting and ensemble mechanisms. To extend the GBM idea to transfer learning, an instance weighting mechanism is added to penalize those source domain instances with a different distribution than the target instances. The instance weights are a function of the power of a base learner and the residual of the instance prediction using the model of the previous step. If an instance of the source domain is misclassified, based on its residual, a weight smaller than 1 is assigned to it so that its effect on the next iteration will reduce. The structure of the presented transfer learning approach is summarized in Figure 1.

The framework of the proposed TrGB model is presented in Algorithm 1. The algorithm is built on top of the original GBM structure with proper modifications to enable transfer learning. As seen, the input data includes the source data (*i* = *1*, …, *m*) and the labeled part of the target data (*i* = *m+1*, …, *n*). Since the loss gradient plays a crucial role in GBM, a differentiable loss function is required. For the binary classification problem, we used negative log-likelihood as the loss function as follows


(1) $$-\sum_{i=1}^{n}y_{i}\,\log\left(p\right)+\left(1-y_{i}\right)\,\text{log}\,\left(1-p\right)$$

**Table:** 

Algorithm 1: TrGB
Input: Labeled source data: ${\left\{\left(x_{i},y_{i}\right)\right\}}_{i=1}^{m}$, and labeled target data ${\left\{\left(x_{i},y_{i}\right)\right\}}_{i=m+1}^{n}$.
A differentiable loss function *L*(*y*_*i*_,*F*(*x*)).
The number of iterations T.
a. Initialize the model as $F_{0}\,\left(\text{x}\right)=\arg m\,i\,n_{\gamma }\,\sum_{i=1}^{n}L\,\left(y_{i},\gamma \right)$
b. For t = 1 to T:
1. Calculate the pseudo-residuals:
$r_{i\,t}=-{\left[\frac{\partial L\,\left(y_{i},F\,\left(x_{i}\right)\right)}{\partial F\,\left(x_{i}\right)}\right]}_{F\,\left(x_{i}\right)=F_{t-1}\,\left(x_{i}\right)}$for i = 1, …, n.
2. Calculate the performance of each weak learner (λ) based on the residuals of target domain data:
${\left[r_{t-a\,v\,g}\right]}_{i=m+1}^{n}$
$\lambda =\frac{1}{2}\,\text{log}\,\left(\frac{1-r_{t-a\,v\,g}}{r_{t-a\,v\,g}}\right)$
3. Calculate weights for both source and target domain instances:
$w_{i\,t}=1\;i\,f\,\left(\frac{1}{2}-r_{i\,t}\right)\,\lambda >0$
$w_{i\,t}=\left\{ \begin{array}{c} e^{-2\,\left(r_{i\,t}-\frac{1}{2}\right)\,\lambda }\,\mathrm{for}\,i=1,\ldots ,m\\ e^{2\,\left(r_{i\,t}-\frac{1}{2}\right)\,\lambda }\,\mathrm{for}\,i=\text{m}+1,\ldots ,n \end{array}\right.\;i\,f\,\left(\frac{1}{2}-r_{i\,t}\right)\,\lambda <0$
4. Fit a base learner (e.g., decision tree) to pseudo-residuals using weighted source and target domain instances ${\left\{w_{i\,t}\,x_{i},r_{i\,t}\right\}}_{i=1}^{n}$ i = 1, …, m (source); i = m+1, …, n (target).
5. Optimizing the following equation and determining the corresponding γ_*m*_:
$\gamma_{m}=\arg m\,i\,n_{\gamma }\,\sum_{i=1}^{n}L\,\left(y_{i},F_{-m\,1}\,\left({\text{x}}_{i}\right)+\gamma \,h_{m}\,\left(x_{i}\right)\right)$
6. Updating the model:
*F*_*m*_(*x*) = *F*_*m*−1_(*x*) + γ_*m*_*h*_*m*_(*x*)

where *y*_*i*_ is the actual class label for the *i*th instance and *p* is the predicted probability. In step a, the model is initialized with a γ value that minimizes the loss function. To do so, the derivative of the loss function is calculated, which is equal to the residuals (*p-y_*i*_*) for the log-likelihood loss function. Thus, for all instances, the initial model would be the mean value of *y*_*i*_ in terms of probabilities and *0.5log((1+y_*avg*_)/(1-y_*avg*_))* in terms of log(odds) as shown in the original GBM algorithm (Friedman, 2001). In the next step, for the first decision tree (*t* = *1*), pseudo-residuals (*r*_*it*_) are calculated for both source and target instances using the gradient of the loss function as follows


(2) $$r_{i\,t}=-{\left[\frac{\partial L\,\left(y_{i},F\,\left(x_{i}\right)\right)}{\partial F\,\left(x_{i}\right)}\right]}_{F\,\left(x_{i}\right)=F_{t-1}\,\left(x_{i}\right)}$$

where *r*_*it*_ is the pseudo-residuals for the *i*th instance and *t*th iteration tree. For the loss function used in this study, pseudo-residuals are simply the difference between the predicted probability and the true values (*y_*i*_-p*). Next, the effectiveness of the *t*th learner denoted by λ is determined as follows


(3) $$\lambda =\frac{1}{2}\,\text{log}\,\left(\frac{1-r_{t-a\,v\,g}}{r_{t-a\,v\,g}}\right)$$

where *r*_*t–avg*_ is the average of the absolute value of residuals over the target data instances. Since λ is defined as a measure of the goodness of a learner, the source domain instances are not included in it. This equation maps the value of *r_*t–avg*_*, which is between [0, 1], into values between [−∞, +∞]. Lower values of *r*_*t–avg*_ close to 0 are associated with large negative values of λ, and values of *r*_*t–avg*_ close to 1, correspond to larger positive values of λ. Thus, a good model will have low *r*_*t–avg*_ and high positive λ.

Using the λ parameter and the residuals, weight coefficients are calculated and assigned to the instances. For a positive value of λ (powerful model) and an instance with a small residual (correctly classified), the weight of the instance will not be changed for the next iteration. However, for a positive value of λ, and a misclassified instance, a different weight is assigned to the instance depending on the fact that the data is from the source or target domain. If the misclassified instance comes from the target dataset, a large weight is given to the instance in order for the model to strengthen its impact on the model for the next iteration. On the other hand, a misclassified instance from the source data is given a lower weight to have a more negligible effect on the model. The weights equation is given below.

$$w_{i\,t}=\{\begin{array}{c} e^{-2\,\left(r_{i\,t}-\frac{1}{2}\right)\,\lambda }\,\mathrm{for}\,i=1,\ldots ,m\\ e^{2\,\left(r_{i\,t}-\frac{1}{2}\right)\,\lambda }\,\mathrm{for}\,i=\text{m}+1,\ldots ,n \end{array}\,i\,f\,\left(\frac{1}{2}-r_{i\,t}\right)\,\lambda <0\,\left(4\right)$$

where *w*_*it*_ is the weight of the *i*th instance in the *t*th iteration. The reasoning for such a weighting mechanism is that a misclassified instance from the source domain is likely to have a different distribution than the target data, and its effect needs to be minimized. The equations in step b.3 of the algorithm implement the described weighting procedure.

The next steps are similar to the original GBM algorithm. First, a base regression learner such as a decision tree is trained using weighted source and target instances as the input data and pseudo-residuals as the target variable. This learner will try to fix the errors (residuals) of the previous tree by minimizing the loss function, as shown in step b.5 of the algorithm. Finally, the model is updated using the new predictions of the regression tree (step b.6). The whole process will be repeated for the next value of *t* until it reaches the maximum value of iterations or until the loss is lower than a predefined threshold.

## Results

To evaluate the effectiveness of our transfer learning framework, a set of experiments was conducted on the Mount Sinai data as the target domain and ADNI as the source domain datasets. The regional cortical thickness from MRI and SUVR values from amyloid PET was used as the feature set for our predictive models. Two single modality scenarios for MRI and amyloid PET and one multi-modality scenario for the combination of MRI and PET have been tested. As for the clinical diagnosis groups, the Mount Sinai dataset includes Normal, PreMCI Clinical, PreMCI Neuropsychology, non-amnestic MCI, Early MCI, Late MCI, and Dementia. In this study, four class labels, including Normal, Early MCI, Late MCI, and Dementia, from the Mount Sinai dataset and three class labels, including Cognitively Normal, MCI, and AD from the ADNI dataset were used. For the label space, two main scenarios were investigated. First, the same label spaces were used for both source and target domains. The purpose of this test was to use the knowledge in the source model for the same classification problem (e.g., CN/MCI, or CN/AD) in the target domain. In the second step, the knowledge was transferred between different label spaces. More specifically, the information from the CN vs. AD classification task from the source domain (ADNI) was used for the Early MCI vs. Late MCI classification in the target domain. As explained, this is one of the scenarios of the transfer learning that is useful here as the amount of labeled data for the Early MCI, and Late MCI groups is too limited for building a model from scratch. In order to report the classification metrics, a 5-fold cross-validation was implemented. Since the datasets are imbalanced, besides accuracy, other evaluation metrics, including Precision, Recall, and F1-score, are also calculated and reported.

For comparison purposes, a baseline case is defined where no information from the source data is used, and a model is built solely based on the labeled data from the target domain. In another case, we assumed that there are no labeled instances from the target domain, and a model is trained on the source data and tested on the target data. In the third case, the TrAdaBoost approach, which is presented in Dai et al. (2007), is used. The final scenario uses the proposed TrGB approach proposed in this study. As a first step, an experiment is conducted on the ADNI dataset without making use of transfer learning. The classification scores for the three tasks of binary and multiclass classification using multimodal data are presented in Table 2. It should be noted that the target data is not included in this experiment. As expected, the most challenging tasks remain the multiclass classification and the binary classification of the CN/MCI cases.”

**TABLE 2:** Classification scores for three classification tasks using the ADNI dataset.

	CN-AD	CN-MCI	CN-MCI-AD
Accuracy	94.5	65.3	56.6
Precision	92.9	59.3	61.1
Recall	91.8	58.7	54.1
F1-score	92.3	58.5	56.6

Tables 3–5 compares the TrGB and baseline cases for the AD diagnosis and for the three modality scenarios. As can be seen, while TrGB could improve the classification scores for all three situations, it has been most effective for the MRI modality case. The classification accuracy of the TrGB is 6.7%, 1.3%, and 2.6% higher than that of the baseline using MRI, PET, and multimodal data, respectively. When using the source model for target testing, the scores are slightly lower than those of the baseline. This situation can be compared with the CN/AD case in Table 2, where the source model was tested on the source data. It can be concluded that the scores of the CN/AD classification using the source model decreased by almost 10% when the target domain was used as the test data. This shows the detrimental effect of data distribution shift between the source and target domains on the classification performance.

**TABLE 3:** CN/AD classification scores for the baseline, source-model, and TrGB scenarios for the MRI modality.

	Baseline	Source-model	TrGB
Accuracy	72.0	70.3	78.7
Precision	70.9	68.7	77.8
Recall	71.1	70.4	78.3
F1-score	71.0	71.3	78.0

**TABLE 4:** CN/AD classification scores for the baselines, source-model, and TrGB scenarios for the PET modality.

	Baseline	Source-model	TrGB
Accuracy	80.0	78.7	81.3
Precision	79.2	78.2	80.8
Recall	78.9	78.1	80.0
F1-score	79.0	78.1	80.3

**TABLE 5:** CN/AD classification scores for the baselines, source-model, TrAdaBoost, and TrGB (proposed) scenarios using the multimodal data.

	Baseline	Source-model	TrAdaBoost	TrGB
Accuracy	82.7	80.0	86.5	85.3
Precision	82.0	79.5	85.3	84.9
Recall	81.7	80.6	83.2	84.4
F1-score	81.8	79.6	84.9	84.6

**FIGURE 2:**
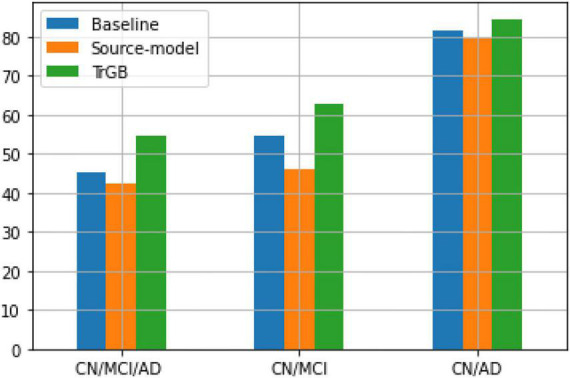
F1-score using the three methods, baselines, source-model, and TrGB for the three classification tasks, CN/MCI/AD, CN/MCI, and CN/AD, using multimodal data.

Tables 6, 7 report the results for the two tasks of MCI and multiclass classification. Again, TrGB could improve the scores compared to the other methods. More specifically, compared to the baseline, the F1-score of the two tasks increased by 8.3 and 9.3%, respectively. It can also be seen that the source model has the worst performance when the MCI subjects are involved in the analysis. This outcome suggests that the MCI group of the two datasets has a more significant discrepancy in terms of marginal and conditional distributions. It can also be observed that the TrAdaBoost approach achieves comparatively acceptable results; however, TrGB yielded higher scores in most cases, especially in the challenging cases of the CN/MCI classification (Table 6) and in the multiclass classification of CN/MCI/AD (Table 7). Figure 2 summarizes the results through F1-scores of the three methods (baseline, source-model, and TrGB) for the three classification tasks considered.

**TABLE 6:** CN/MCI classification scores for the baselines, source-model, TrAdaBoost, and TrGB scenarios using the multimodal data.

	Baseline	Source-model	TrAdaBoost	TrGB
Accuracy	75.6	75.2	75.9	77.1
Precision	60.3	42.5	63.1	65.9
Recall	54.9	50.0	59.7	61.7
F1-score	54.6	46.0	61.4	62.9

**TABLE 7:** CN/MCI/AD classification scores for the baselines, source-model, TrAdaBoost, and TrGB scenarios using the multimodal data.

	Baseline	Source-model	TrAdaBoost	TrGB
Accuracy	57.4	50.1	61.2	61.9
Precision	56.5	41.2	56.3	60.9
Recall	43.8	39.4	50.1	51.9
F1-score	45.2	42.6	52.9	54.5

Finally, an experiment for the early MCI vs. late MCI classification in the target domain was conducted by transferring the knowledge from the CN vs. AD classification of the source domain (Table 8). The TrGB algorithm could enhance the performance of this task compared to the baseline. The results prove the effectiveness of the proposed transfer learning model for challenging classification tasks where the two classes are very similar and the number of labeled instances is limited.

**TABLE 8:** Early MCI vs. late MCI classification scores for the baselines and TrGB scenarios using the multimodal data.

	Baseline	TrGB
Accuracy	57.4	60.4
Precision	46.9	53.2
Recall	47.9	52.8
F1-score	46.6	52.6

While the proposed transfer learning approach can boost the classification performance for a target dataset, there are some limitations associated with it. First, this approach increases the model complexity, which requires more training time and resources. Also, since the model is based on GBM, the process of creating base learners is sequential, which is computationally intensive. For faster implementations and superior performance, alternative algorithms, including Extreme Gradient Boosting (XGB) and Light Gradient Boosting Machine (Light GBM), could be used. Another important circumstance in transfer learning techniques is negative transfer, where the transferred information from the source domain has a detrimental effect on the performance of the target model. In the proposed model, the defined weighting scheme aims to transfer as much information as possible from the source domain while avoiding negative transfer at the same time. Thus, selecting appropriate weights is essential to make a balance between these two conflicting requirements.

## Conclusion

In this study, a novel transfer learning framework based on the gradient boosting machine (TrGB) was proposed. The aim was to use knowledge from an auxiliary dataset for a classification task in a target dataset with insufficient training data for learning a model. Like with the GBM algorithm, in TrGB, the base learners focus on the residual of their previous learners. In addition, the residuals are used to assign a weight to the source and target instances such that misleading instances of the source domain have a more negligible effect on the learning process. The experimental results show that the TrGB achieved superior performance for MCI and AD classification compared to the baseline case, where a model is trained only using the labeled instances of the target domain. It was shown that the MCI group has more data distribution shift between the two datasets than the AD group. Transfer learning was also beneficial for the early MCI vs. late MCI classification in the target dataset using the knowledge in the CN vs. AD classification model of the source domain.

## Data availability statement

The original contributions presented in this study are included in the article. Further inquiries on data availability can be directed to the corresponding author or MA.

## Ethics statement

This study was conducted according to Good Clinical Practice guidelines, US 21CFR Part 50—Protection of Human Subjects, and Part 56—Institutional Review Boards (IRBs)/Research Ethics Boards (REBs), and pursuant to state and federal HIPAA regulations. The patients/participants provided their written informed consent to participate in this study.

## Author contributions

MS designed and developed the original model and performed the analysis and interpretation of the data and results. MA provided overall guidance on the model’s design and helped with the analysis and interpretation of the results. MC helped with the data analysis. MS and MA wrote the manuscript. SD, DV, DL, and RD helped with the editing and provided clinical input on the transfer learning approach. All authors revised the manuscript for scientific content and final approval.

## Footnotes

1
http://adni.loni.usc.edu
